# Prescriptions

**Published:** 1895-03

**Authors:** 


					TONIC.
R. Tinct. ferri chloridi, . . i.
Acid phosphor, dil., . .  i.
Syr. limonis,.....................%ii.
M. Sig.Teaspoonful in water after meals.
MALARIA, CHRONIC.
R. Acid carbolic, . . . gr. xiij. Tinct. iodi., .... 3 i. Syrupi,..........................3xxiij
M. Sig.  Teaspoonful three times daily, in water, as an adjunct to quinine.
DIARRHCEA.
R. Aq. camphorae, . . iij. Tr. lavender comp., .  i.
Tr. opii,..................' 3i to ij.
M. Sig.Teaspoonful every hour or two.
By i)r. Barthlow.

SORE NIPPLES.
R. Aq. rosae, .........................Jij.
Glycerinae,..........................%ij.
Acid tannici,.....................3ij-
M. Sig.Apply on lint.
DIPHTHERIA.
R. 01. eucalypti, . . . . 3 ij - Sodii benzoatis, . . . . j.
Glycerini,..........................$ij.
Aquae calcis, q. s. a. d., . Oij.
M. Sig.Apply as a spray to the, throat every half hour.
By Dr. Wilson.
R. Acidi salicylici, . . . . 3iv.
Spts. vini rect, . . . . 5 iv.
Chloroformi,................3iv.
Tinct. opii, ..................3 v.
01. dulcis, q. s. a. d., . .
M. Sig.Use as liniment.
 By Dr. Bevillod.

Prescription Department.
BOTKINS DROPS FOR CHOLERA.
In Russia, they use a mixture known as Botkins drops very extensively (from the name of an eminent physician, who popularized the prescription).
B. Liq. anod. Hoffmanii.
Tr. cinchonae co., . . aajj. Quiniae, mur., .... 3 j-
Acidi, mur., dil., . . . 3Jss.
01. menth.,..................gr. ss.
M. Sig.  Teaspoonful every hour until relief.
FOR DIPSOMANIA.
B. Tinct Capsici . . . M. x. Sod. Bromid. .... gr. x. Spt. Ammon. Aromat. . 3 j.
M. To be taken in a little water three times a day.N. Y. Medical .Tmtrnnl
FOR ERYSIPELAS.
B. Tannin......
Camphor . . . . aa 1 part.
Ether ...... 8 parts.
M. To be applied with a brush three times a da j.Ex.
pruritus.
Dr. Goodell prescribes the following for pruritus:
B. Hydrargyri Chlorid.
Corros.....................gr. j.
Pulv. Aluminis . gr. xx.
Amyli . . . . . 3 iss.
Aquae.........................3 vj.
M. Sig.Apply locally.
NERVOUS HEADACHE.
B. Potass. Bromid . 4drms.
Liq. Tong.'Sal . 8 ozs.
M. ft. sol. Sig.Teaspoonful
every hour until the desired effect is secured.



				

